# Integrating DNA methylation measures to improve clinical risk assessment: are we there yet? The case of *BRCA1* methylation marks to improve clinical risk assessment of breast cancer

**DOI:** 10.1038/s41416-019-0720-2

**Published:** 2020-02-18

**Authors:** Ee Ming Wong, Melissa C. Southey, Mary Beth Terry

**Affiliations:** 1 Precision Medicine, School of Clinical Sciences at Monash Health, Monash University, Clayton, VIC Australia; 2 Department of Clinical Pathology, The University of Melbourne, Melbourne, VIC Australia; 3 Cancer Epidemiology Division, Cancer Council Victoria, Melbourne, VIC Australia; 4 Department of Epidemiology, Mailman School of Public Health, Columbia University, New York, NY USA; 5 Herbert Irving Comprehensive Cancer Center, Columbia University, New York, NY USA

**Keywords:** Cancer epigenetics, Breast cancer, Methylation analysis

## Abstract

Current risk prediction models estimate the probability of developing breast cancer over a defined period based on information such as family history, non-genetic breast cancer risk factors, genetic information from high and moderate risk breast cancer susceptibility genes and, over the past several years, polygenic risk scores (PRS) from more than 300 common variants. The inclusion of additional data such as PRS improves risk stratification, but it is anticipated that the inclusion of epigenetic marks could further improve model performance accuracy. Here, we present the case for including information on DNA methylation marks to improve the accuracy of these risk prediction models, and consider how this approach contrasts genetic information, as identifying DNA methylation marks associated with breast cancer risk differs inherently according to the source of DNA, approaches to the measurement of DNA methylation, and the timing of measurement. We highlight several DNA-methylation-specific challenges that should be considered when incorporating information on DNA methylation marks into risk prediction models, using *BRCA1*, a highly penetrant breast cancer susceptibility gene, as an example. Only after careful consideration of study design and DNA methylation measurement will prospective performance of the incorporation of information regarding DNA methylation marks into risk prediction models be valid.

## Background

Current cancer risk prediction models are largely grouped based on the extent of family history and genetic data in addition to information relating to non-genetic risk factors such as lifestyle and the environment. Pedigree-based models, which are often used for genetic counselling and risk assessment and for making decisions about chemoprevention and risk-reducing surgeries,^1^ have been improved through the integration of genetic information about high and intermediate penetrant cancer genes.^2–4^ Three such breast cancer models—BOADICEA (Breast and Ovarian Analysis of Disease Incidence and Carrier Estimation Algorithm model), IBIS (the International Breast cancer Intervention Study model) and BRCAPRO—have the ability to predict the probability of carrying a pathogenic breast cancer susceptibility variant in *BRCA1/2* as well as the absolute risk of developing breast cancer, with higher discrimination for the former than the latter.^2–4^ BOADICEA has also been extended to include information about genetic variation in *CHEK2*, *PALB2* and *ATM*,^5^ as well as polygenic risk scores (PRS), which include hundreds of genetic variants identified from large genome-wide association studies (GWAS).^6,7^ Although the gain from inclusion of a PRS may be limited overall, there is gain in prediction for women at the extremes of the PRS. In contrast to BOADICEA, IBIS and BRCAPRO, which includes details about the ages of cancer diagnoses in the relatives as well as germline mutations in BRCA1/2, other breast cancer risk models like the commonly used Breast Cancer Risk Assessment Tool (BCRAT) generally consider family history based on the number of first-degree family relatives affected and/or ever/never family history. Most models now include non-genetic established breast cancer risk factors (e.g. parity, age at menarche, hormone use).

Increasingly large-scale studies support that many of the breast cancer predisposition genes can also influence risk when altered through epigenetic mechanisms, such as DNA methylation and histone modification, and that such mechanisms might occur more commonly than currently appreciated.^8,9^ Epigenetic alterations have been demonstrated to be associated with an increased risk of certain diseases, such as Fragile X syndrome, Prader–Willi syndrome, and various cancers, and are increasingly being measured in epidemiological studies.^10–12^ However, potential integration of epigenetic information into risk prediction models requires further prospective evidence that might be more challenging to collect than those studies incorporating genetic information.

Here, we outline the case for the integration of epigenetic measures, in the form of blood-based DNA methylation marks, into existing risk prediction models using the example of *BRCA1*, the breast cancer susceptibility gene with the highest penetrance, to illustrate the gaps in our knowledge that need to be addressed to improve clinical risk assessment. We selected this gene because the integration of germline mutation status of *BRCA1* has already been shown to improve risk assessment and knowledge of the germline *BRCA1* status has already altered clinical practice in terms of chemoprevention, recommendations for risk-reducing surgeries, and screening frequency. We consider germline DNA methylation of *BRCA1* in all women and not just in women with pathogenic variants in *BRCA1*. The points we raise would also relate to other epigenetic marks such as histone acetylation and histone methylation.

DNA methylation refers to the addition of a methyl (CH_3_) group to the cytosine residue of a cytosine-guanidine pair in the DNA sequence, commonly referred to as a CpG dinucleotide. DNA methylation is an essential component in early development through a process known as epigenetic reprogramming.^13^ In adult cells, it has been shown to be extensively involved in the initiation and progression of cancer whereby aberrant DNA methylation can lead to silencing and loss of expression of tumour suppressor genes such as *BRCA1*, *MLH1* and *ATM*^14–17^ and genomic instability.^18,19^ In breast cancer, aberrant DNA methylation levels across specific breast cancer susceptibility genes, such as *BRCA1*,^20–22^ *ATM*,^15,23^ *PALB2*^24^ and *Sat2*,^25^ have been associated with risk of the disease in women with and without pathogenic variants in these genes.

Given that high and intermediate penetrant germline mutation data are now key components to selected risk models like BOADICEA, the potential for improved model performance when extending risk models to include epigenetic markers in the same genes may be great. Several important areas need to be considered before integrating DNA methylation marks into risk assessment models, including the source and nature of the biological material, the approaches used to measure DNA methylation (methodologies and regions of DNA), and the timing of the biological sample collection for DNA methylation measurement, all of which are discussed below using *BRCA1* as an example.

## Biological material for DNA methylation assessment

Early studies focused on identifying changes in DNA methylation marks in disease-affected tissues^26,27^ have demonstrated the utility of these changes in further subtyping cancers and refining precision medicine,^28–30^ as well as proving valuable for predicting prognosis after cancer diagnosis.^31–33^ The use of DNA methylation marks for risk prediction, however, often requires the use of surrogate tissue and/or blood-based biomarkers (for review, see refs. ^9,34^) that can be reliably and repeatedly measured using non-invasive sampling. Here we would like to emphasise that a good predictive marker need not be measured from the potential site of carcinogenesis, e.g. measured in DNA sourced from breast tissue or breast milk to determine breast cancer risk—a good predictive marker needs only to be associated with the disease of interest and to be stable over repeated measurements.

Caution is rightly warranted when using blood-derived DNA modifications as biomarkers. DNA methylation displays cell-type-specific heterogeneity^35^ and, as such, methylation measured in blood-derived DNA is influenced by the proportion of cell types present in the blood sample. To address this, study designs often match case–control pairs by the source of DNA (e.g. whole blood, lymphocyte fraction, buffy coat) and control for variation in blood sample cellularity as part of the analytic process using statistical methods such as that proposed by Houseman et al.^36^ Continued improvement of these statistical methods will further improve the accuracy of cell-type adjustment. Specifically, white blood cells, as a non-invasive source of DNA prior to disease onset, has been used for studies searching for DNA methylation marks that can be useful for understanding cancer susceptibility (the focus of this Perspective).

Using DNA derived from peripheral blood, we reported that constitutional *BRCA1* promoter methylation—that is, DNA methylation that is present in every cell of the body—is associated with a 3.5-fold increased risk (95% confidence interval [CI]: 1.4, 10.5) of developing early-onset breast cancer of a specific histological type in non *BRCA1* mutation carriers.^21^ Another study conducted in Japan also reported that *BRCA1* promoter methylation detected in peripheral blood cells is associated with an increased risk of developing breast cancer (all ages) (odds ratio [OR] 1.73, 95% CI: 1.01, 2.96).^20^ Methylation of the *ATMmvp2a* intergenic region and the *Sat2* repetitive element have also been reported to be associated with an increased risk of breast cancer (women in the highest quintile OR 1.89, 95% CI: 1.36, 2.64 in peripheral blood, and OR 2.09, 95% CI: 1.09, 4.03 in white blood cells, respectively).^23,25^ Xu et al.^10^ identified 250 blood-based CpG dinucleotides that were differentially methylated (*P*_FalseDiscoveryRate_ < 0.05) between cases and controls,^10^ and that five differentially methylated CpG dinucleotides had similar model discrimination as the BCRAT model that included nine GWAS common variants (area under the curve (AUC) 65.8%, 95% CI: 61.0, 70.5% versus 66.1%, 95% CI: 61.0, 71.3%).

Several epigenome-wide association studies (EWAS) have also demonstrated an association between global DNA methylation levels and breast cancer risk.^11,12^ However, a 2019 meta-analysis of four EWAS did not find an association between blood-based DNA methylation and the risk of breast cancer;^37^ this result could perhaps be partly explained by the later age of onset in the affected women (further discussed below), or the different analytic approaches used by each study (e.g. cell-type correction, normalisation of raw data and data transformation).

## DNA methylation: methodology and measurement

Laboratory methodologies vary and thus measures of DNA methylation cannot, in all instances, be directly compared or easily combined.^9,34^ We will not discuss these methods in detail except to outline in Box 1 the experimental throughput, sensitivity, input DNA requirements and cost—issues that should be considered for epidemiology studies and when evaluating data for integration into cancer risk models. Laboratory methods used to assess DNA methylation can be divided into three main categories: loci-specific; array-based and bisulphite sequencing methods (see Box 1).

Given the different considerations for each of the three methodologies, an approach that targets risk-associated DNA methylation regions, akin to the commonly used gene panel sequencing methods in the germline context, might prove the most useful in integrating new information into cancer risk models. For example, although identified GWAS variants may be used in the future clinically through an integration of a PRS, genetic information from sequencing studies, which include fuller genetic alterations than those included in GWAS, are already being used clinically through gene panel tests. In a similar way, we might expect that studies that include deeper investigation of epigenetic alterations in the same genes used in these gene panels may be clinically more beneficial than information from an EWAS, which only includes selected CpGs from these high and intermediate penetrant genes.

A meta-analysis found that DNA methylation of the *BRCA1* promoter was more common in women diagnosed with breast cancer compared with unaffected women.^38^ A number of different study designs with different methylation markers, including methylation measured in blood-derived DNA and from histologically normal and malignant breast tissues, were included in this meta-analysis. *BRCA1* promoter methylation was associated with an increased risk of developing breast cancer (OR 3.15, 95% CI: 1.97, 5.03, *P* < 0.001), advanced stage histopathology features, and triple-negative disease.^38^ When considering only the nine blood-based studies, *BRCA1* methylation was associated with a 1.87-fold increased breast cancer risk (95% CI: 1.19, 2.96, *P* = 0.007).^38^

Tang et al.^34^ published a comprehensive review of blood-derived DNA methylation marks associated with an increased risk of breast cancer. Although overall increased *BRCA1* promoter methylation was observed in the blood-derived DNA of affected women, the breast cancer risk estimates of the meta-analysis showed considerable variation between studies.^34^ While the factors mentioned above (DNA source, measurement type, methodology) could have contributed to the observed heterogeneity, it is possible that different regions measured even within the same gene, as highlighted by Zhang and Long,^38^ could also provide some explanation for the variation.

**Fig. 1:**
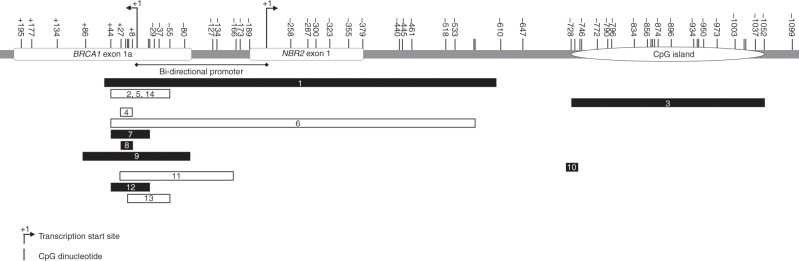
The *BRCA1* promoter regions assessed for methylation by the studies listed in Table 1. The number(s) in each bar corresponds to the study number (#) in Table 1. The *BRCA1* promoter region assessed by each study is represented by a horizontal bar. Black bars represent studies that measured *BRCA1* promoter methylation in blood-derived DNA. White bars represent studies that measured *BRCA1* promoter methylation in blood-derived DNA and DNA derived from sources other than blood. Each CpG dinucleotide is represented as a vertical line and numbered relative to the *BRCA1* transcription start site (denoted by +1).

Although the majority of studies have assessed blood-derived DNA methylation in the same *BRCA1* promoter region (primarily overlapping the bi-directional promoter and transcription start sites), some studies have measured DNA methylation at other loci, such as the CpG island, which is likely to have contributed to the different findings (Table 1; Fig. 1).^39,40^ Our experience with the Infinium HumanMethylation450 BeadChip array, which has been validated using TCGA data, showed that the CpG island (Chromosome17: 41278135–41278459) is highly methylated in blood-derived DNA from both affected and unaffected women and lacks variability between individuals.^41,42^ Therefore, DNA methylation assessment of this invariant region is likely to be uninformative and, indeed, no differences in *BRCA1 *DNA methylation levels were observed in this region between cases and controls irrespective of their *BRCA1* or *BRCA2* mutation status.^39,40^ Ziller et al.^43^ found that DNA methylation levels across the intermediate and low CpG density promoters and transcription start sites, rather than the CpG islands, are dynamic and variable between individuals.^42,43^ These two observations strongly indicate that the region outside of the CpG island, rather than the CpG islands itself, could stand to be more informative for DNA methylation assessment for risk prediction.

**Table 1:** Published studies on *BRCA1* promoter methylation in breast and/or ovarian cancer.

#	Reference	Methodology	Region^a^	DNA source	Study groups	Age (years)	Findings
1	Chen et al. (2006)^44^	Bisulfite cloning Sanger sequencing	−591 to +66	Whole blood	Affected *BRCA1* and *BRCA2* mutation-negative women with a strong family history (*n* = 41) Unaffected women (*n* = 19)	20–50	No significant difference in *BRCA1* methylation levels of individual CpGs between affected and unaffected women
2	Snell et al. (2008)^69^	MethyLight Real-time PCR	+14 to +16	Whole blood	Women diagnosed with *BRCA1*-like BC (*n* = 7)	35–51	One women was methylated at the *BRCA1* promoter in her DNA derived from blood and buccal mucosa (10% and 5%, respectively). Two women had ~1% *BRCA1* methylation
		MS-HRM Digital MS-HRM Sanger sequencing	−55 to +44	Buccal mucosa Breast tumour	Corresponding buccal mucosa DNA (*n* = 1) Tumour DNA (*n* = 5)		Tumour-derived DNAs from all three women had high levels of *BRCA1* methylation (61–100%)
3	Kontorovich et al. (2009)^39^	MS-qPCR Sanger sequencing	−728 to −1052	Whole blood	Affected *BRCA1/2* mutation carriers (*n* = 48) Unaffected *BRCA1/2* mutation carriers (*n* = 41) Affected non-mutation carriers (*n* = 52) Healthy controls (*n* = 89)	26–62	Promoter methylation only detected in *BRCA1* (5.6–7.3% in each study group) *BRCA1* promoter methylation detected in ~5% of the Israeli Jewish women regardless of the *BRCA1*/*2* status Proportion with *BRCA1 *promoter methylation according to age (all groups): <40 years: 9/118 (7.6%) 41–50 years: 4/68 (5.9%) 51–60 years: 1/27 (3.7%) ≥61 years: 0/17 (0%)
4	Cho et al. (2010)^80^	MethyLight Real-time PCR	+8 to +27	Breast tumour Adjacent normal tissue White blood cells	BC patients (*n* = 40) Healthy controls (*n* = 40)	< 40–> 60	Most patients with methylation at *BRCA1*, *HIN1*, *RASSF1A* and *CDH1* in their blood-derived DNA were also methylated in their corresponding tumour-derived DNA Gene-specific methylation in blood-derived DNA the same in cases and controls
5	Wong et al. (2011)^21^	MS-HRM MethyLight	−55 to +44 +14 to +16	Whole-blood Breast tumour	Group 1: Young women diagnosed with ‘*BRCA1*-like’ BC (*n* = 52) Group 2: Young women diagnosed with non ‘*BRCA1*-like’ BC (*n* = 164) Unaffected women (*n* = 169)	< 40	*BRCA1* promoter methylation detected in blood-derived DNA of 30% Group 1 women compared with Group 2 (*P* = 0.000002) and unaffected women (*P* = 0.004). *BRCA1* promoter methylation associated with rs11655505 C > T (*P* = 0.035) and rs799906 A > G (*P* = 0.017). *BRCA1 *promoter methylation in blood-derived DNA was associated with a 3.5-fold increased risk of developing ‘*BRCA1*-like’ BC (95% CI: 1.4, 10.5).
6	Al-Moghrabi et al. (2011)^64^	MSP High-resolution sodium bisulfite sequencing	−37 to +44 –567 to +44	Breast tumour Whole blood	Tumour-derived DNA from affected Arab women (*n* = 47) Blood-derived DNA from other women diagnosed with BC (*n* = 7) Healthy controls (*n* = 73)	22–80	Strong association between *BRCA1* methylation and young age (≤40 years) at diagnosis and high-grade tumours (67%) (*P* = 0.0038) *BRCA1* promoter methylated in blood-derived DNA of 8/73 healthy controls (10.9%) and 2/7 BC patients (28%) *BRCA1* CpG island highly methylated in blood-derived DNA in both cases and controls Sporadic *BRCA1* methylation detected in the core promoter region (−218 to +1)
7	Wojdacz et al. (2011)^67^	MS-HRM	−21 to +44	Whole blood	Affected women (*n* = 180) Unaffected women (*n* = 108)	> 50	No significant difference in frequency of *BRCA1* methylation between cases (20%) and controls (20%)
8	Iwamoto et al. (2011)^20^	qMSP	+8 to +27	Whole blood	Affected women (*n* = 200) Unaffected women (*n* = 200)	30–69	*BRCA1* promoter methylation detected in blood-derived DNA of 21.5% (43/200) affected women and 13.5% (27/200) unaffected women OR_adj_ for BC is 1.73; 95% CI: 1.01, 2.96; *P* = 0.045)
9	Hansmann et al. (2012)^22^	Pyrosequencing Bisulfite plasmid sequencing Promoter sequencing	−116 to +116	Whole blood	Affected women negative for *BRCA1* and *BRCA2* mutations (*n* = 641)	< 40–>60	1.4% (9/641) patients had *BRCA1* methylation (6–20%). *BRCA1* epimutations were (i) Significantly more frequent in index patients with OC than BC (*P* = 0.016) (ii) More frequent in families with BC and OC history than with BC alone (*P* = 0.01) (iii) Significantly enriched in women with early-onset BC without a family history of cancer, compared with women with familial BC after the age of 36 years (*P* = 0.047)
10	Bosviel et al. (2012)^40^	QAMA	−662 to −678	Whole blood	Mutation-negative affected women (*n* = 902) Unaffected women (*n* = 990)	26–89	*BRCA1* promoter methylation rate was 47.1% (95% CI: 46.1, 48.1) in affected women and 45.9% (95% CI: 45.0, 46.8) in unaffected women (*P* = 0.08) In blood-derived DNA of affected women, *BRCA1* promoter methylation was associated with (i) Age (*P* = 0.017) (ii) Post-menopausal status (*P* = 0.013) (iii) BMI < 20 (*P* = 0.0095) (iv) WHR (≤76.8) (*P* = 0.0027) *BRCA1* promoter methylation was not associated with Scarff-Bloom-Richardson tumour grade and size, histological type and lymph node metastasis
11	Al-Moghrabi et al. (2014)^66^	MSP	−156 to +34	White blood cell Breast tumour	Affected women (*n* = 155) Unaffected women (*n* = 143) Tumour tissues from 22 methylation-positive patients (*n* = 19)	23–73	14.2% (22/155) affected women (20 were <50 years old) and 9.1% (13/143) unaffected women (11 were <40 years old) had *BRCA1 *promoter methylation in their blood DNA 66.7% affected women had *BRCA1* promoter methylation in blood-derived and tumour-derived DNA *BRCA1* methylation was higher in cases than controls (corresponding lower mRNA expression)
12	Gupta et al. (2014)^65^	MS-HRM	−21 to +44	Whole blood	Affected *BRCA1*-mutation carriers (*n* = 30) Affected non carriers (*n* = 36) Unaffected women (*n* = 36)	> 60 (mean age)	*BRCA1* promoter methylation present in 41.7% (15/36) affected mutation-negative women but not affected mutation carriers (*P* < 0.01) *BRCA1* methylation-positive women tend to be younger (average 42.5 years) OR for BC in affected mutation-negative women with detectable *BRCA1* methylation was 12.1 (95% CI: 2.5, 59.0) *BRCA1* methylation more common in TNBC [40.9% (9/22)], medullary BC [45.4% (10/22)] and both triple-negative and medullary BC [50% (4/8)]
13	Cho et al. (2015)^68^	MethyLight Real-time PCR	−55 to +16	WBC Breast tumour	Population-based cases (*n* = 1021) Population-based controls (*n* = 1036) Tumour tissue (*n* = 569)	< 40–> 75	*BRCA1* hypermethylation in WBC associated with an increased risk of BC (≥0.1% methylation (OR 1.31; 95% CI: 0.98, 1.75)) Lack of concordance between tumor tissue and paired WBC DNA methylation Limited evidence for *BRCA1* methylation in WBC DNA and BC risk
14	Evans et al. (2018)^45^	Pyrosequencing Bisulfite sequencing	−55 to +44	Lymphocyte Blood Buccal mucosa Hair follicle Breast tumour	Lymphocyte: Unrelated individuals from high-risk BC and OC families (*n* = 49) Blood: Family 1 (*n* = 10); Family 2 (*n* = 4) Buccal mucosa: Family 1 (*n* = 9); Family 2 (*n* = 4) Hair follicle: Family 1 (*n* = 9); Family 2 (*n* = 4) Breast tumour: Family 1 (*n* = 1); Family 2 (*n* = 0)	31–85	*BRCA1 *promoter methylation detected in: (i) 2/49 women diagnosed with early-onset BC (lymphocyte and blood) (ii) Six family members in Family 1. 2/6 diagnosed with early-onset BC (iii) Four family members in Family 2. 1/4 diagnosed with early-onset OC (iv) associated with a 5’UTR dominantly inherited c.−107A > T variant No correlation between methylation levels and clinical phenotype The epimutation accounts for at least 1.25% *BRCA1* pathogenic variants in high-risk BC and OC families

The methodology, region interrogated, DNA source, age of participants, study groups and findings for each study are as listed. # denotes the study number corresponding to Fig. 1

*BC* breast cancer, *MethyLight* MethyLight Real-time PCR, *MS-HRM* methylation-specific High Resolution Melt analysis, *MSP* methylation-specific PCR, *OC* ovarian cancer, *QAMA* Quantitative Analysis of Methylated Alleles, *WBC* white blood cell

^a^Relative to BRCA1 transcription start site

Only three CpG dinucleotides in *BRCA1* exon 1a (+8, +14 and +16) have been evaluated across most studies (12 of the 14 studies listed in Table 1; Fig. 1). However, variability in DNA methylation levels and analysis outcomes across these three sites could still be possible for several reasons as highlighted below. A key aspect that may affect interpretation of the differences across studies is whether or not the study has specifically sampled women with a breast cancer family history, and with few exceptions,^44^ most studies of the association between epigenetic marks and breast cancer risk have been conducted in mainly post-menopausal women. Additionally, inconsistencies in the evidence could be explained by the different methodologies used to assess DNA methylation: seven different methods were used across the 14 studies (Table 1). With the exception of pyrosequencing, bisulphite cloning followed by Sanger sequencing or bisulphite sequencing, methylation-specific methods such as Methylation-Sensitive High Resolution Melt (MS-HRM) analysis and MethyLight real-time PCR, provide an average methylation value across the interrogated region. Unless all CpG dinucleotides in the region of interest display similar methylation levels, this average methylation value might not enable an association between methylation and breast cancer risk, particularly if only a subset of CpG dinucleotides in the region are relevant to disease risk.

Differences across studies might also be driven by genetic variation, which can influence DNA methylation levels at some genomic regions (known as methylation quantitative trait loci). We previously identified two *BRCA1 *promoter region genetic variants in blood-derived DNA that were associated with *BRCA1* promoter methylation (rs11655505 (*P* = 0.035) and rs799906 (*P* = 0.017)),^21^ but the publication of few, if any, reports on genetic variants that are positively associated with *BRCA1* promoter methylation rendered these results inconclusive until Evans et al.^45^ identified a dominantly inherited *BRCA1* 5’ untranslated region variant (NM_007294.3:c.−107 A > T) in two multiple-case breast and ovarian cancer families.^45^ Hemi-methylation of the *BRCA1* promoter region was found soma-wide, including in leukocyte-derived DNA in heterozygote carriers with *BRCA1 *promoter methylation associated with the c.−107 A > T variant, explaining at least 1.25% of *BRCA1* pathogenic variants in their multiple-case breast and ovarian cancer families (Table 1).

Box 1 Laboratory methods to assess DNA methylation Loci-specific methods include MethyLight Real-time PCR,^46^ Methylation-Specific PCR,^47^ EpiTYPERMassARRAY System^48^ and pyrosequencing.^49^ Although capable of detecting low levels of methylation (as low as 1%), these methods are laborious and low-throughput (limited by the number of samples and/or number of regions that can be evaluated in any one assay). Individual assays need to be designed for each region of interest, with each amplicon typically 100–200 base pairs (bp) in size to retain its sensitivity (500 bp for the MassARRAY). DNA of variable quality derived from blood, plasma, dried blood spots and formalin-fixed paraffin-embedded (FFPE) material can be applied to these methods.^21,25^ Loci-specific assays are cost-effective and their increased sensitivity makes them suitable for validation studies.

Array-based methods that measure DNA methylation at a large number of CpG dinucleotides across the genome are less sensitive than loci-specific methods particularly at the extremes of DNA methylation^50–54^ although the sensitivity of the Illumina Methylation EPIC array seems to have improved markedly at these extreme DNA methylation levels.^52^ Arrays are suitable candidates for large epidemiology studies and have been instrumental in the success of a large number of EWAS.^11,12,37^ These arrays specifically assess the most informative regions of the epigenome such as gene promoters, CpG islands, enhancer and regulatory regions. Although relatively cost-effective in the context of throughput and number of evaluated CpG dinucleotides, further validation is often required due to their limited sensitivity. DNA derived from blood, plasma, dried blood spots, saliva, fresh-frozen and FFPE material have all been successfully assayed using this platform.^42,55,56^

Bisulphite sequencing methods include reduced-representation bisulphite sequencing (RRBS),^57^ MethylC-seq,^58^ targeted bisulphite sequencing and whole-genome bisulphite sequencing (WGBS).^59^ These methods quantitatively detect methylation at single base resolution and, depending on the assay, can evaluate methylation at a specific region (e.g. targeted bisulphite sequencing), at CpG-dense areas (e.g. RRBS) or across the methylome(e.g. WGBS).The increased resolution of these techniques also enables the detection of technical variabilities not previously possible using lower resolution techniques. The community’s experience with WGBS is mixed: areas of variable coverage and bisulphite conversion inefficiencies within and between, samples have, in many instances, affected data interpretation. It should also be noted that, of the 28 million CpG dinucleotides across the methylome, 70–80% are stably methylated and therefore are uninformative across different cell types.^43^ Bisulphite sequencing methods are the most expensive of the laboratory methods and generally require high molecular weight DNA, which can be challenging for many epidemiology studies that only have access to limited quantities of often degraded DNA derived from field-collected bioresources. Additionally, as sample size increases, the common set of CpGs with quality data after an experiment may result in a matrix containing similar or fewer CpGs than an array-based approach.

## Timing of sample collection for DNA methylation measurement

Measuring DNA methylation marks requires considerable attention to the hypothesis being tested to not only define the relevant target tissue, but to also ascertain the appropriate timing of the biological sample collection. Blood-based DNA methylation marks can be altered by intrauterine exposures (e.g. prenatal famine^60^) as well as environmental conditions later in life (e.g. air pollution^61,62^). Alterations in DNA methylation might hold great potential as surrogate markers for factors that are not easily measured by questionnaires—for instance, Boyne et al.^62^ and Johansson et al.^63^ have found that long-term hormonal exposure can be proxied through DNA methylation signatures.

However, although the potential for the use of DNA methylation markers is great, there are also many challenges that need to be overcome for DNA methylation measures to make a significant contribution to breast cancer risk prediction models. The key considerations include making the appropriate measurements and then interpreting these measurements, especially those that depend on time cognisant of the research question. Conducting GWAS and measuring common genetic variation in large consortia, by contrast, have generally been straightforward, as genes do not change with time, DNA can be collected from a variety of sources, and the type of genotyping assay does not usually affect the aggregating of data. However, exposure measurements in large consortia are often limited to a single time point, and repeated measurements of lifestyle and environmental exposures are rarely available. Unfortunately, this also applies to studies trying to identify DNA methylation risk factors for disease, which may be just as important if not more so than genetic (GWAS-discovered) variants in the same gene.^10–12^

Changes in DNA methylation over time can also affect the interpretation of data collected across different participant age groups. In Table 1, studies include individuals in different age groups, with the larger studies weighted towards older, average-risk individuals. Associations between *BRCA1* DNA methylation and breast cancer have more commonly been reported by studies that specifically examined *BRCA1 *promoter methylation in younger women (< 45 years) or stratified their findings by age at diagnosis.^21,22,64–66^ When we specifically sampled for early-onset breast cancer cases (< 40 years), the association with *BRCA1 *promoter methylation and breast cancer risk is much stronger (OR 3.5, 95% CI: 1.4,10.5).^21^ Wodjacz et al.^67^ measured *BRCA1* methylation in post-menopausal women and failed to find any significant differences between cases and controls. Cho and colleagues^68^ assessed *BRCA1 *promoter methylation in women across a wide age range (< 45 to > 75 years old) but did not consider a sub-analysis of young women, which is particularly relevant as *BRCA1 *promoter methylation is more frequently detected in young women diagnosed with breast tumours and with a specific histological type.^21,66,69^ If the breast cancer risk associated with *BRCA1* promoter methylation mimics germline pathogenic variants (where the incidence of breast cancer in *BRCA1* mutation carriers rises rapidly after the age of 30 years^70^) the timing of DNA sampling and the case mix in terms of age and absolute risk in studies of DNA methylation will likely affect the relative risk estimates for methylation markers and breast cancer.

Finally, epigenetic aging should also be taken into account (biological age based on the methylation measurement of specific sets of CpG dinucleotides) as it has been shown to be associated with risk of mortality and disease.^71–73^ Using a prospective study design and three epigenetic clocks (Horvath, Hannum and Levine),^74–76 ^Kresovich  et al.^77^ found that a 5-year acceleration of epigenetic age (defined as the difference between the biological age and chronological age) was associated with increased risk for breast cancer (hazard ratio [HR]: 1.08–1.15, 95% CI: 1.00–1.23, *P* < 0.001–0.04). The inclusion of data on epigenetic age acceleration might therefore be pertinent to increase the accuracy of breast cancer risk prediction models.

## Conclusions

Extensive efforts to identify DNA methylation marks associated with risk of breast cancer have so far identified a small number of potential DNA methylation marks of interest with the prime example being *BRCA1*. We have outlined a number of issues for consideration before information about DNA methylation can be integrated into breast cancer risk prediction models. We used the example of *BRCA1* to illustrate the challenges faced when considering DNA methylation changes in a breast cancer susceptibility gene, as well as to caution that even when considering single-gene studies, results can be heterogeneous depending on study selection, sampling and laboratory methods.

With management of the above concerns, this line of research has several strengths, including the prevalence of DNA methylation changes (which is often higher than that of genetic changes), the potential magnitude of the association between DNA methylation and cancer risk, and DNA methylation alterations reflecting in part changes in exposures and conditions during the course of life. These three factors combined suggest that overcoming the challenges in conducting studies and implementing measurements in the clinic might be outweighed by the gain in improved accuracy of risk prediction models and ultimately the more precise identification of individuals who can benefit most from early intervention and early detection. As an example, there is an increasing incidence of advanced breast cancer in young women under the age of 40 in the USA^78^ and, globally, breast cancer remains the top cancer in terms of both incidence and mortality in women in most countries across continents^79^— additional methods are needed to identify these high-risk women. It is in this context that measures of DNA methylation, particularly for DNA methylation alterations in high and intermediate penetrant breast cancer susceptibility genes, combined with germline genetic testing of cancer susceptibility genes might identify young women who are at increased risk with more precision than current approaches and models. Women identified as being at high risk via these multi-omic approaches might benefit from supplemental screening modalities such as MRI. Incorporation of information regarding DNA methylation changes, including those that target high and intermediate penetrant breast cancer susceptibility genes, into risk assessment may therefore prove important in ages and settings where population-based screening by mammography is not possible.

## Data Availability

No data were generated for this manuscript.
